# Biomarkers of Oncogenesis, Adipose Tissue Dysfunction and Systemic Inflammation for the Detection of Hepatocellular Carcinoma in Patients with Nonalcoholic Fatty Liver Disease

**DOI:** 10.3390/cancers13102305

**Published:** 2021-05-11

**Authors:** Gian Paolo Caviglia, Angelo Armandi, Chiara Rosso, Silvia Gaia, Serena Aneli, Emanuela Rolle, Maria Lorena Abate, Antonella Olivero, Aurora Nicolosi, Marta Guariglia, Davide Giuseppe Ribaldone, Patrizia Carucci, Giorgio Maria Saracco, Elisabetta Bugianesi

**Affiliations:** 1 Department of Medical Sciences, University of Turin, 10126 Torino, Italy; angelo.armandi@unito.it (A.A.); chiara.rosso@unito.it (C.R.); serena.aneli@unito.it (S.A.); marialorena.abate@unito.it (M.L.A.); antonella.olivero@unito.it (A.O.); aurora.nicolosi999@edu.unito.it (A.N.); marta.guariglia@edu.unito.it (M.G.); davidegiuseppe.ribaldone@unito.it (D.G.R.); giorgiomaria.saracco@unito.it (G.M.S.); 2 Gastroenterology Unit, A.O.U. Città della Salute e della Scienza, 10126 Torino, Italy; sgaia2@cittadellasalute.to.it (S.G.); erolle@cittadellasalute.to.it (E.R.); pcarucci@cittadellasalute.to.it (P.C.); 3 Department of Biology, University of Padua, 35122 Padova, Italy

**Keywords:** adiponectin, AFP, GPC-3, HCC, IL-6, leptin, NAFLD, PIVKA-II, surveillance

## Abstract

**Simple Summary:**

Circulating biomarkers for the detection of hepatocellular carcinoma in patients with dysmetabolic liver disease are an unmet need. In the present study, we observed that serum values of five biomarkers (namely, AFP, PIVKA-II, GPC-3, adiponectin and IL-6) were significantly different between patients with and without hepatocellular carcinoma; the best accuracy for the detection of tumor was achieved by PIVKA-II. Furthermore, we developed a model combining age, gender, PIVKA-II, GPC-3 and adiponectin that showed an excellent performance for the identification of patients with hepatocellular carcinoma. This model may be useful for the surveillance of patients with dysmetabolic liver disease at risk of hepatocellular carcinoma development.

**Abstract:**

Current surveillance strategy for patients with nonalcoholic fatty liver disease (NAFLD) at risk of hepatocellular carcinoma (HCC) development is unsatisfactory. We aimed to investigate the diagnostic accuracy of alpha-fetoprotein (AFP), protein induced by vitamin K absence or antagonist-II (PIVKA-II), glypican-3 (GPC-3), adiponectin, leptin and interleukin-6 (IL-6), alone or in combination, for the discrimination between NAFLD patients with or without HCC. The biomarkers were investigated in a cohort of 191 NAFLD patients (median age 62, 54–68 years; 121 males and 70 females) with advanced fibrosis/cirrhosis, 72 of whom had a diagnosis of HCC. PIVKA-II showed the best performance for the detection of HCC with an area under the curve (AUC) of 0.853, followed by adiponectin (AUC = 0.770), AFP (AUC = 0.763), GPC-3 (AUC = 0.759) and by IL-6 (AUC = 0.731), while the leptin values were not different between patients with and without HCC. The accuracy of the biomarkers’ combination was assessed by a stratified cross-validation approach. The combination of age, gender, PIVKA-II, GPC-3 and adiponectin further improved the diagnostic accuracy (AUC = 0.948); the model correctly identified the 87% of the patients. In conclusion, we developed a model with excellent accuracy for the detection of HCC that may be useful to improve the surveillance of NAFLD patients at risk of tumor development.

## 1. Introduction

The epidemiological burden of nonalcoholic fatty liver disease (NAFLD) is rapidly increasing worldwide, with an estimated global prevalence of 25% in the general population [1]. NAFLD includes a broad spectrum of liver diseases, ranging from simple steatosis to nonalcoholic steatohepatitis (NASH), a condition at risk of progression to liver cirrhosis and hepatocellular carcinoma (HCC) [2]. In addition, the coexistence of multiple metabolic risk factors such as obesity and type 2 diabetes mellitus (T2DM) can synergistically promote tumorigenesis in patients with NAFLD and advanced liver disease [3].

Adipose tissue is recognized as endocrine organ able to produce several adipokines, such as adiponectin and leptin, involved in the regulation of metabolism and inflammation [4]. Adipokines dysregulation has been associated with systemic low-grade inflammation [5], impaired hepatocyte proliferation [6] and progression from NASH to HCC [7]. In particular, interleukin-6 (IL-6) can activate the signal transducer and activator of transcription 3 (STAT3) signaling [8], a major intrinsic pathway involved in cell proliferation, migration and survival [2,9].

Currently, surveillance programs for patients at risk for HCC development are based on ultrasound (US) screening at 6-month intervals, while the combined use of serum biomarkers is still a matter of debate [10,11,12,13]. However, US screening is limited by the poor sensitivity for the detection of small liver nodules [14,15]; besides, NAFLD-related HCC may develop in the absence of liver cirrhosis [16], hampering the efficacy of current surveillance strategies mainly aimed at patients with cirrhosis. Therefore, novel noninvasive tools able to improve the surveillance of patients with NAFLD are urgently needed.

Among the traditional HCC biomarkers, protein induced by vitamin K absence or antagonist II (PIVKA-II), alone or in combination with alpha-fetoprotein (AFP), showed promising results for the early detection, and even for the prediction, of HCC in patients with cirrhosis of viral etiology [17,18,19,20]. However, the data on NAFLD-related HCC are scant. Glypican-3 (GPC-3) is an oncofetal protein normally not expressed in the livers of healthy adults; so far, the available data on the performance of serum GPC-3 for HCC detection are conflicting and mainly confined to patients chronically infected with the hepatitis B virus (HBV) or hepatitis C virus (HCV) [21].

To date, the early detection of HCC represents the major goal in order to improve patients’ survival; thus, the identification of high-performing biomarkers able to promptly identify patients with HCC among patients at risk of tumor development is an unmet need. Here, we investigated the diagnostic accuracy of selected tumor biomarkers (i.e., AFP, PIVKA-II and GPC-3); adipokines (i.e., adiponectin and leptin) and IL-6, alone or in combination, for the discrimination between patients with or without NAFLD-related HCC.

## 2. Materials and Methods

### 2.1. Patients

This retrospective case-control study included patients with dysmetabolic induced-HCC and patients with NAFLD/NASH without HCC, recruited at the outpatient clinic of the Unit of Gastroenterology of A.O.U. Città della Salute e della Scienza di Torino—Molinette Hospital, Turin, Italy between November 2012 and January 2020.

For all patients, the inclusion criteria were age ≥18 years, histological diagnosis of NASH with advanced fibrosis/cirrhosis or clinical/radiological evidence of cryptogenic cirrhosis [22], which dysmetabolic etiology was assessed by the presence of metabolic risk factors (central obesity, T2DM, dyslipidemia and hypertension) [23] in the absence of other known causes of liver damage. For patients without HCC, a minimum of 1-year US follow-up after the collection of the serum sample was required. All patients signed written informed consent.

We excluded patients with a liver disease of other etiology, such as drug-induced liver disease, viral hepatitis and autoimmune, cholestatic and metabolic/genetic liver disease. Alcohol-induced liver disease was excluded by selecting patients with a negative history of alcohol abuse (weekly ethanol consumption <140 g for women and <210 g for men) [24].

The presence of advanced fibrosis/cirrhosis was histologically assessed and scored as described by Kleiner et al. [25] or clinically determined by means of a liver elastography (FibroScan^®^, Echosens™, Paris, France) or hepatic US features and endoscopic signs of portal hypertension [26,27]. The diagnosis of HCC was achieved by histological examination or by contrast-enhanced imaging methods showing the radiological hallmark of HCC (i.e., the combination of hypervascularity in the late arterial phase and washout on portal venous and/or delayed phases), following the international guidelines [11]. HCC was classified according to the BCLC staging system (0 = very early, A = early, B = intermediate, C = advanced and D = terminal stage) [11].

### 2.2. Measurement of Circulating Biomarkers

Serum and plasma samples were collected in polypropylene 2-mL tubes labeled with the study participant identification code and stored at −80 °C until analysis. Serum levels of AFP, PIVKA-II, adiponectin and IL-6 were determined on the fully automated chemiluminescent enzyme immunoassay (CLEIA) system, LUMIPULSE G600 II analyzer (Fujirebio Inc., Tokyo, Japan) using Lumipulse^®^ G AFP-N (assay precision <3%), Lumipulse^®^ G PIVKA-II (assay precision <4.4%), Lumipulse^®^ G HMW Adiponectin (assay precision ≤4%) and IL-6 LPG reaction cartridges, according to the manufacturer’s instructions. The lower limit of detection was 0.075 ng/mL for AFP, 1.37 mAU/mL for PIVKA-II, 0.09 µg/mL for adiponectin and 0.2 pg/mL for IL-6.

Serum GPC-3 and plasma leptin values were measured by enzyme-linked immunosorbent assay (ELISA) using CanAg Glypican-3 EIA (Fujirebio Diagnostics AB, Gothenburg, Sweden) and Human Leptin Quantikine^®^ ELISA (R&D Systems, Minneapolis, MN, USA), according to the manufacturer’s instruction. The GPC-3 serum levels were reported in pg/mL, while the leptin plasma levels were reported in ng/mL.

### 2.3. Statistical Analysis

Continuous variables were expressed as median and interquartile ranges (IQR), while categorical variables as number and percentages (%). The D’Agostino-Pearson test was used to test the data normality. The Mann–Whitney test and Kruskal–Wallis test were used to compare continuous variables between two or more groups, respectively. The correlation between continuous variables was assessed by Spearman’s rank correlation coefficient (*r_s_*). To evaluate the diagnostic accuracy of the circulating AFP, PIVKA-II, GPC-3, adiponectin, leptin and IL-6 alone, the AUC was assessed by receiver operating characteristic (ROC) curves analysis. A multivariate logistic regression analysis was performed to combine independent variables for the prediction of HCC. This analysis has been repeated using a cross-validation approach to compute its performances in predicting the HCC status, using the *scikit-learn* package in the Python environment. Specifically, the *RepeatedStratifiedKFold* with 5 splits and shuffling samples 20 times and the *LogisticRegression* functions were used to evaluate the performance of the classifier using a cross-validation approach, both with the option random_state = 0. The final performance of the model were computed, averaging the AUC values over the 100 test sets obtained with the above-described cross-validated approach. Confidence intervals at a 95% confidence level were computed with a bootstrap approach by resampling with a replacement 1000 times.

A two-tailed *p* < 0.05 was considered statistically significant. The statistical analyses were performed using MedCalc software, version 18.9.1 (MedCalc bvba, Ostend, Belgium) and in-house scripts in Python programming language.

## 3. Results

### 3.1. Patients’ Characteristics

A total of 191 patients (median age 62, 54–68 years; male (M) = 121 and female (F) = 70) were included in the study. The demographic, clinical and biochemical characteristics of the study population are reported in Table 1.

Patients with HCC (*n* = 72) were older than patients without tumor (*n* = 119) (67, 62–70 years vs. 58, 49–66 years, *p* < 0.001) and had a higher prevalence of males (*n* = *57*, 79% vs. *n* = *64*, 54%, *p* = 0.010). No differences were observed in the prevalence of obesity (*p* = 0.450), T2DM (*p* = 0.764), dyslipidemia (*p* = 0.086) and hypertension (*p* = 1.000). The majority of patients were cirrhotic (*n* = 129, 68%), with a higher prevalence of cirrhosis in patients with HCC compared to those without tumors (88% vs. 55%, *p* < 0.001). Consistently, patients with HCC showed significantly higher values of total bilirubin (*p* = 0.001) and INR (*p* < 0.001) and lower values of platelets (*p* < 0.001) and albumin (*p* < 0.001). Among patients without HCC, 95 had a histological diagnosis of NAFLD/NASH (F3 = 53, F4 = 42), while 24 had a clinical diagnosis of cirrhosis. Among the patients with HCC, the diagnosis of tumor was achieved by pathology in 14 patients, by multiphasic computed tomography (CT) in 35 patients and by dynamic contrast-enhanced magnetic resonance imaging (MRI) in 23 patients. Overall, 47 patients had a diagnosis of early tumor (BCLC 0 = 7, A = 40) and 22 of advanced tumor (BCLC B = 15, C = 7), while three patients had terminal-stage HCC (BCLC D = 3).

### 3.2. Circulating Biomarkers Values in the Study Cohort

**Figure 1:**
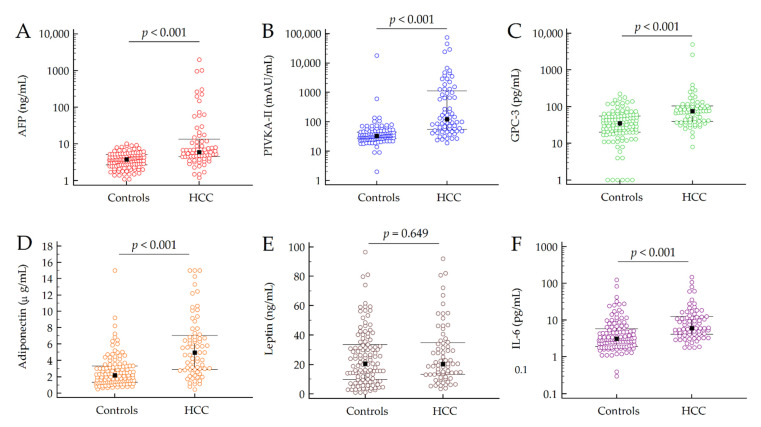
Median values of AFP (**A**), PIVKA-II (**B**), GPC-3 (**C**), adiponectin (**D**), leptin (**E**) and IL-6 (**F**) in patients with and without HCC. *P*-values were calculated by Mann–Whitney test. Black squares and error bars represent, respectively, the median value and the IQR in each group of patients. The values of AFP, PIVKA-II, GPC-3 and IL-6 are depicted in Log scale due to data skewness. Abbreviations: alpha-fetoprotein (AFP), glypican 3 (GPC3), hepatocellular carcinoma (HCC) and interleukin-6 (IL-6).

The median serum levels of AFP, PIVKA-II, GPC-3, adiponectin and IL-6 were significantly higher in patients with HCC compared to those without tumor (all *p* < 0.001); only the plasma leptin values were not different between the two groups of patients (*p* = 0.649) (Table 2 and Figure 1).

The AFP serum values showed a significant stepwise increase from patients with advanced fibrosis without HCC to patients with advanced tumor (BCLC = B, C and D) (*p* < 0.001). The AFP and PIVKA-II levels were significantly different between the patients with early HCC (BCLC = 0, A) and those with advanced tumor (*p* < 0.050), while among patients without HCC, the AFP, GPC3 and IL-6 serum levels were significantly different between the patients with advanced fibrosis and those with cirrhosis (*p* < 0.050) (Table S1 and Figure S1).

**Figure 2:**
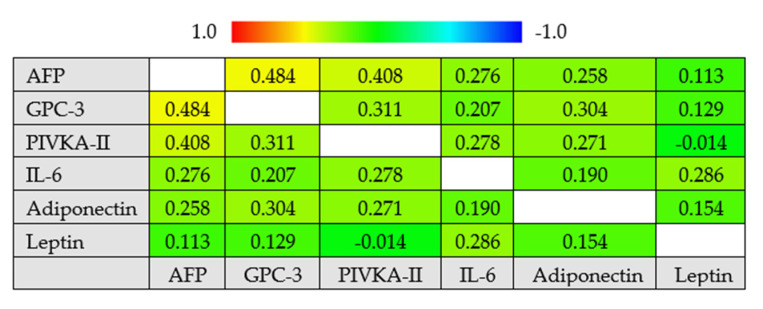
Correlogram of the biomarkers’ concentrations. Cells are colored according to the magnitude of the correlations, ranging from dark red for positive correlations to dark blue for negative correlations. Correlation coefficients (*r_s_*) were calculated by the Spearman test. Abbreviations: alpha-fetoprotein (AFP), glypican 3 (GPC3), hepatocellular carcinoma (HCC) and interleukin-6 (IL-6).

By the Spearman correlation analysis, we observed that the serum AFP values were moderately correlated to PIVKA-II (*r_s_* = 0.408, 95% confidence interval (CI) 0.283–0.520, *p* < 0.001) and to the GPC-3 serum levels (*r_s_* = 0.484, 95% CI 0.368–0.586, *p* < 0.001). PIVKA-II was moderately correlated to GPC-3 (*r_s_* = 0.311, 95% CI 0.177–0.434, *p* < 0.001), and GPC-3 was moderately correlated to serum adiponectin (*r_s_* = 0.304, 95% CI 0.169–0.428, *p* < 0.001) (Figure 2).

### 3.3. Diagnostic Accuracy of Circulating Biomarkers for HCC Detection

**Figure 3:**
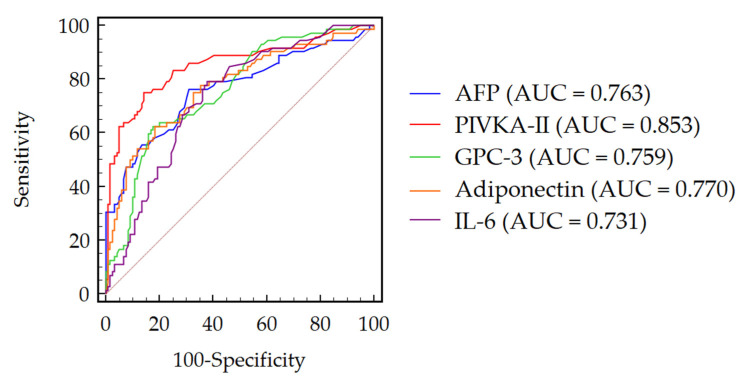
ROC curves of AFP, PIVKA-II, GPC-3, adiponectin and IL-6 discrimination between patients with and without HCC. Abbreviations: alpha-fetoprotein (AFP), area under the curve (AUC), glypican 3 (GPC-3), interleukin-6 (IL-6) and protein induced by vitamin K absence or antagonist II (PIVKA-II).

The diagnostic accuracy of AFP, PIVKA-II, GPC-3, adiponectin and IL-6 for the discrimination between patients with and without HCC was assessed by the receiver operating characteristic (ROC) curve analysis. The values of the area under the curve (AUC), sensitivity (Se), specificity (Sp), positive likelihood ratio (+LR) and negative likelihood ratio (−LR) are reported in Table 3. PIVKA-II showed a higher performance with AUC = 0.853, followed by adiponectin (AUC = 0.770), AFP (AUC = 0.763), GPC-3 (AUC = 0.759) and by IL-6 (AUC = 0.731) (Figure 3). Similar results were observed, following the stratification for the BMI and T2DM (Figure S2). By ROC curve comparison, the diagnostic accuracy for HCC detection of PIVKA-II was significantly superior to the performance of AFP (∆AUC = 0.090, *p* = 0.044), GPC-3 (∆AUC = 0.094, *p* = 0.035) and IL-6 (∆AUC = 0.122, *p* = 0.009), while only a trend was observed for the comparison between PIVKA-II and adiponectin (∆AUC = 0.083, *p* = 0.075). No other significant differences were observed from the comparison between AFP, GPC-3, adiponectin and IL-6 (Table S2). Finally, we performed a sub-analysis to investigate the biomarkers accuracy for the detection of early tumors (BCLC = 0 and A); PIVKA-II showed the higher performance (AUC = 0.810), followed by GPC-3 (AUC = 0.749), adiponectin (AUC = 0.744), AFP (AUC = 0.704) and by IL-6 (AUC = 0.699) (Figure S3).

### 3.4. Predictors of HCC and Model Development

Since the two groups of patients (i.e., patients with and without HCC) showed significant differences regarding the demographic, biochemical and clinical features, we performed a multivariate logistic regression analysis to assess the strength of the association with HCC. Age, gender, BMI, ALT, γGT, platelet count, albumin, total bilirubin, INR, cholesterol, triglycerides, AFP, PIVKA-II, GPC-3, adiponectin and IL-6 were considered for inclusion in the multivariate analysis. A logistic regression analysis was based on a stepwise approach keeping the variables at a significance level below 0.01 [28]. A Log transformation was made to AFP, PIVKA-II, GPC-3 and IL-6 due to data skewness. The variables retained in the model are reported in Table 4.

The obtained formula of the model was:

y = −17.33 + 0.09 × Age + 2.48 × Gender + 1.93 × Log PIVKA-II + 2.75 × Log GPC-3 + 0.43 × adiponectin,

where age in years, 1 for males and 0 for females; the probability (*p*) of HCC is given by:

p = 1 / (1 + e^−y^).

**Figure 4:**
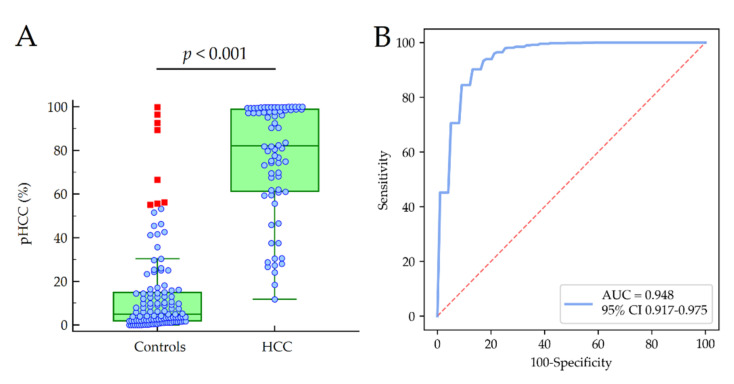
Median values of pHCC in patients with and without HCC (**A**) and diagnostic accuracy of the model (**B**). Red squares indicate values that are larger than the upper quartile plus 3 times the interquartile range. *p*-values were calculated by the Mann–Whitney test. Abbreviations: area under the curve (AUC), confidence interval (CI), hepatocellular carcinoma (HCC) and probability of hepatocellular carcinoma (pHCC).

The median pHCC values were 5.0% (95% CI 3.5–8.3) and 82.2% (95% CI 75.0–96.5) in patients with and without HCC, respectively (*p* < 0.001). The model showed an excellent diagnostic accuracy for the detection of HCC (AUC = 0.948), with a percentage of the patients correctly classified as 87% in the cross-validation (Figure 4). At the cut-off pHCC = 50%, the model showed Sp = 88.1%, Se = 86.9%, +LR = 9.00 and −LR = 0.15 for the detection of HCC.

## 4. Discussion

In the present study, we investigated the performances of different biomarkers involved in the oncogenic mechanisms of HCC in patients with NAFLD. Indeed, the biomarkers studied were selected on the premise that tumor development in such patients is driven by the concurrent activation of different oncogenic signaling pathways in accordance with the “multiple hits hypothesis” [29,30], whereby comorbidities, genetic determinants and environmental factors simultaneously contribute to NAFLD/NASH-HCC progression [31]. Interestingly, we observed that circulating biomarkers such as PIVKA-II and adiponectin displayed a good performance for the discrimination between patients with HCC and those without tumors; remarkably, the combination of demographic features (i.e., age and gender) together with oncogenic markers (i.e., PIVKA-II and GPC-3) and markers of adipose tissue dysfunction (i.e., adiponectin) allowed the development of a model showing an excellent performance for the detection of HCC.

Several different biomarkers have been studied in the last decades in order to improve and even personalize the surveillance of patients at high risk for HCC development [32,33,34]. Promising results have derived from comprehensive approaches that have allowed the detection of a wide spectrum of circulating molecules, including tumor proteins and nucleic acids (i.e., circulating tumor DNA/RNA) derived from the primary tumor [35], epigenetic biomarkers such as DNA methylation profiles and noncoding RNAs [36,37] and genetic variants recapitulated in polygenic risk scores [38]. On the other hand, the study of serologic biomarkers such as AFP and PIVKA-II has been pursued over time due to their inexpensiveness, analytical standardization, and acceptable performances [17].

In agreement with previous studies performed in patients with NAFLD [39,40], we observed a good diagnostic accuracy for PIVKA-II (AUC = 0.853) and a moderate performance for AFP (AUC = 0.763). Interestingly, GPC-3 showed a higher accuracy compared to the results of our previous study carried out on a cohort of patients with viral related-HCC [18]. Possibly, both the diverse etiology and clinical characteristics of the patients included may have accounted for the discrepancy observed. In particular, the different prevalence of cirrhosis in the control groups could have affected the performance of GPC-3. Indeed, cirrhosis is a preneoplastic condition characterized by genetic, epigenetic and molecular alterations not yet established in patients with chronic hepatitis but frequently observed in HCC [41,42,43]. As a matter of fact, the more we reduce the clinical differences between two groups of patients, the more the performance of the biomarker decreases.

Noteworthy, adiponectin showed a performance similar to AFP for the detection of HCC. A recent meta-analysis including nine studies and a total of 705 HCC patients and 1390 healthy controls showed that higher adiponectin levels were significantly associated with liver cancer (standard mean difference = 0.97, 95 %CI 0.02∼1.93, *p*  <  0.05) [44]. Furthermore, in patients with HCV-related cirrhosis, higher serum adiponectin levels resulted in a predictor of HCC development [45,46] and liver-related mortality [47]. However, the mechanism by which adiponectin is involved in HCC development is not fully clear. Reduced adiponectin levels have been associated with metabolic syndrome [4,48]; nevertheless, studies in vitro and in the animal model have revealed an antiproliferative activity for adiponectin [49,50], suggesting that increased adiponectin levels might have a protective role in HCC. Consonant with this hypothesis, the administration of adiponectin (5 µg/kg weekly) blocked tumor progression in a thioacetamide-induced rat HCC model, resulting in an 80% increased survival rate, 73% reduced average number of nodules and 46% decreased serum AFP [51]. Further studies are needed to confirm the hepatoprotective and chemoprotective effects of adiponectin against HCC.

Finally, the combination of different biomarkers with clinical and/or demographic characteristics into simple prediction models allowed the further improvement of the diagnostic accuracy for HCC detection [52]. However, the majority of these models have been developed and validated in cohorts of patients chronically infected with HBV or HCV [28,53,54,55]; to the best of our knowledge, only the GALAD score was tested in the setting of NAFLD, showing a high performance for the identification of patients with HCC [56]. Here, we developed a model including age, gender, PIVKA-II, GPC-3 and adiponectin that showed a high diagnostic accuracy in cross-validation (AUC = 0.948) for the detection of HCC in patients with dysmetabolic liver disease; the model allowed to correctly identify 87% of the patients included in the study. Given the relatively small number of patients enrolled and the lack of a validation cohort, we applied a machine learning approach based on a stratified cross-validation to assess the performance of the model; accordingly, the original sample was partitioned into a training set to train the model and a test set to evaluate its performance, and the procedure was repeated multiple times. As a result, the model denoted a high accuracy, with a low risk of overfitting, and a generalizability of the independent datasets. However, further multicenter studies are needed to independently validate these findings. Furthermore, a cost-effective analysis may be useful to assess the benefits produced by the implementation of the model in the clinical setting with respect to its cost.

## 5. Conclusions

Our data confirmed the good diagnostic accuracy of PIVKA-II for the detection of HCC in patients with NAFLD. Furthermore, the combination of age, gender, PIVKA-II, GPC-3 and adiponectin allowed a noticeable improvement in the detection of HCC compared to each single biomarker used alone. These findings need to be validated in a prospective surveillance setting in order to assess the ability of the model to predict the HCC occurrence in patients with dysmetabolic liver disease at risk of tumor development.

## Figures and Tables

**Table 1:** Characteristics of the patients included in the study according to the diagnosis of HCC.

Characteristics	Controls	HCC	*p*-Value
Patients, *n*	119	72	
Age (years), median (IQR)	58 (49–66)	67 (62–70)	<0.001
Male gender, *n* (%)	64 (54%)	57 (79%)	<0.001
BMI (kg/m^2^), median (IQR)	30.8 (28.0–34.0)	30.4 (26.0–31.7)	0.013
Obesity (BMI ≥ 30.0 kg/m^2^), *n* (%)	72 (61%)	39 (54%)	0.450
T2DM, *n* (%)	68 (57%)	43 (60%)	0.764
Dyslipidemia, *n* (%) *	41 (57%)	83 (69%)	0.086
Hypertension, *n* (%)	88 (74%)	54 (75%)	1.000
ALT (U/L), median (IQR)	53 (31–72)	34 (24–41)	<0.001
AST (U/L), median (IQR)	41 (30–57)	38 (30–50)	0.280
γGT (U/L), median (IQR)	63 (43–120)	87 (57–179)	0.023
Platelets (× 10^9^/L), median (IQR)	194 (161–239)	132 (85–183)	<0.001
Albumin (g/dL), median (IQR)	4.1 (3.8–4.4)	3.9 (3.3–4.1)	<0.001
Total Bilirubin (mg/dL), median (IQR)	0.8 (0.6–1.0)	0.9 (0.7–1.5)	0.001
INR, median (IQR)	1.05 (1.00–1.11)	1.14 (1.09–1.31)	<0.001
Total Cholesterol (mg/dL), median (IQR)	175 (157–203)	168 (139–181)	0.025
HDL-Cholesterol (mg/dL), median (IQR)	46 (39–59)	44 (35–54)	0.368
Triglycerides (mg/dL), median (IQR)	124 (94–160)	115 (84–132)	0.009
Fasting Glucose (mg/dL), median (IQR)	110 (92–129)	112 (100–137)	0.143
Cirrhosis, *n* (%)	66 (55%)	63 (88%)	<0.001
BCLC Stage (0/A/B/C/D), *n*		7/40/15/7/3	
HCC nodules (1/2/3/>3), *n*		33/16/8/15	
Size of major nodule (mm), median (IQR)		18 (15–24)	

* Total cholesterol ≥200 mg/dL and/or HDL cholesterol <40 mg/dL for men and <50 mg/dL for women and/or triglycerides ≥150 mg/dL. *p*-values for the quantitative variables were calculated by Mann–Whitney test, while *p*-values for categorical variables were calculated by Fisher’s Exact test. Abbreviations: alanine aminotransferase (ALT), aspartate aminotransferase (AST), Barcelona Clinic Liver Cancer (BCLC), body mass index (BMI), gamma-glutamyl transpeptidase (γGT), hepatocellular carcinoma (HCC), high-density lipoprotein (HDL), international normalized ratio (INR), interquartile range (IQR), low-density lipoprotein (LDL), number (*n*) and type 2 diabetes mellitus (T2DM).

**Table 2:** Median biomarker levels according to the presence of HCC.

Biomarkers	Controls	HCC	*p*-Value
AFP (ng/mL), median IQR	3.8 (2.7–5.1)	6.0 (4.5–13.5)	<0.001
PIVKA-II (mAU/mL), median IQR	33 (27–45)	121 (54–1135)	<0.001
GPC-3 (pg/mL), median IQR	35 (20–56)	75 (40–104)	<0.001
Adiponectin (µg/mL), median IQR	2.17 (1.35–3.30)	4.95 (2.87–7.03)	<0.001
Leptin (ng/mL), median IQR	20.6 (9.8–33.4)	20.3 (13.2–34.9)	0.649
IL-6 (pg/mL), median IQR	3.1 (1.9–5.8)	6.0 (4.1–12.5)	<0.001

*p*-values were calculated by the Mann–Whitney test. Abbreviations: alpha-fetoprotein (AFP), glypican 3 (GPC3), hepatocellular carcinoma (HCC) and interleukin-6 (IL-6).

**Table 3:** Diagnostic accuracy of AFP, PIVKA-II, GPC-3, adiponectin and IL-6 discrimination between the patients with and without HCC.

Biomarker	AUC, 95%CI	Cut-off *	Se	Sp	+LR	−LR
AFP (ng/mL)	0.763, 0.696–0.821	>4.4	76.4	68.9	2.46	0.34
PIVKA-II (mAU/mL)	0.853, 0.794–0.900	>56	75.0	85.7	5.25	0.29
GPC-3 (pg/mL)	0.759, 0.691–0.817	>64	62.5	82.4	3.54	0.46
Adiponectin (µg/mL)	0.770, 0.704–0.828	>3.68	62.5	81.5	3.38	0.46
IL-6 (pg/mL)	0.731, 0.662–0.792	>3.6	79.2	62.2	2.09	0.34

* Identified by the Youden *J* statistic. AUC values were calculated by the receiver operating characteristic curve analysis. Abbreviations: alpha-fetoprotein (AFP), area under the curve (AUC), confidence interval (CI), glypican-3 (GPC-3), hepatocellular carcinoma (HCC), interleukin-6 (IL-6), protein induced by vitamin K absence or antagonist II (PIVKA-II), sensitivity (Se), specificity (Sp), positive likelihood ratio (+LR) and negative likelihood ratio (−LR).

**Table 4:** Multivariate analysis for the factors associated to HCC.

Variables	OR, 95% CI	*p*-Value
Age (years)	1.09 (1.02–1.16)	0.007
Male gender	11.95 (3.48–41.04)	<0.001
PIVKA-II (Log mAU/mL)	6.87 (2.03–23.23)	0.002
GPC-3 (Log pg/mL)	15.63 (2.99–81.59)	0.001
Adiponectin (µg/mL)	1.54 (1.23–1.94)	<0.001

Abbreviations: confidence interval (CI), glypican-3 (GPC-3), hepatocellular carcinoma (HCC), odds ratio (OR) and protein induced by vitamin K absence or antagonist II (PIVKA-II).

## Data Availability

The data presented in this study are available upon request from the corresponding author.

## Supplementary Materials

The following are available online at https://www.mdpi.com/article/10.3390/cancers13102305/s1: Figure S1: Median values of AFP (A), PIVKA-II (B), GPC-3 (C), adiponectin (D), leptin (E) and IL-6 (F), according to the different stages of liver disease, Figure S2. Diagnostic accuracy of AFP, PIVKA-II, GPC-3, adiponectin and IL-6 for the detection of HCC in lean (A) and obese patients (B), and in normal-glucose tolerant (C) and diabetic patients (D), Figure S3. Diagnostic accuracy of AFP, PIVKA-II, GPC-3, adiponectin and IL-6 for the detection of early HCC, Table S1: Median biomarker values according to the different stages of liver disease, Table S2: Comparison of the diagnostic accuracy of AFP, PIVKA-II, GPC-3, adiponectin and IL-6 for the detection of HCC.
