# Supplementary material for: Biomarkers of Oncogenesis, Adipose Tissue Dysfunction and Systemic Inflammation for the Detection of Hepatocellular Carcinoma in Patients with Nonalcoholic Fatty Liver Disease
Source: Cancers (Basel). 2021 May 11;13(10):2305. doi: 10.3390/cancers13102305 (PMC8151983; doi:10.3390/cancers13102305)
Supplement: Supplementary file 1 [file cancers-13-02305-s001.zip › cancers-1197881-supplementary.pdf]

cancers

MDPI

Supplementary Materials

Table S1. Median biomarkers values according to the different stages of liver disease.

Biomarkers	Controls		HCC		*p*-Value
	Advanced Fibrosis	Cirrhosis	Early (BCLC = 0/A)	Advanced (BCLC = B/C/D)	
AFP (ng/mL), median IQR	3.0 (2.0–3.8)	4.2 (3.5–5.4)	5.0 (3.7–8.6)	7.8 (5.7–36.7)	<0.001
PIVKA-II (mAU/mL), median IQR	33 (27–47)	33 (28–44)	74 (45–204)	658 (145–2144)	<0.001
GPC-3 (pg/mL), median IQR	27 (17–40)	43 (20–71)	73 (39–101)	80 (40–108)	<0.001
Adiponectin (μg/mL), median IQR)	1.79 (1.21–3.01)	2.42 (1.47–3.54)	4.64 (2.64–6.62)	5.54 (3.03–10.11)	<0.001
Leptin (ng/mL), median IQR	15.5 (5.9–28.3)	26.3 (15.4–37.7)	19.5 (13.5–31.2)	20.4 (9.4–56.6)	0.020
IL-6 (pg/mL), median IQR	2.9 (1.8–4.6)	3.3 (2.5–7.0)	5.8 (3.8–11.3)	9.5 (4.6–18.5)	<0.001

p values were calculated by Kruskal-Wallis test. Abbreviations–alpha-fetoprotein (AFP), Barcelona Clinic Liver Cancer (BCLC), glypican 3 (GPC3), hepatocellular carcinoma (HCC), interquartile range (IQR), interleukin-6 (IL-6).

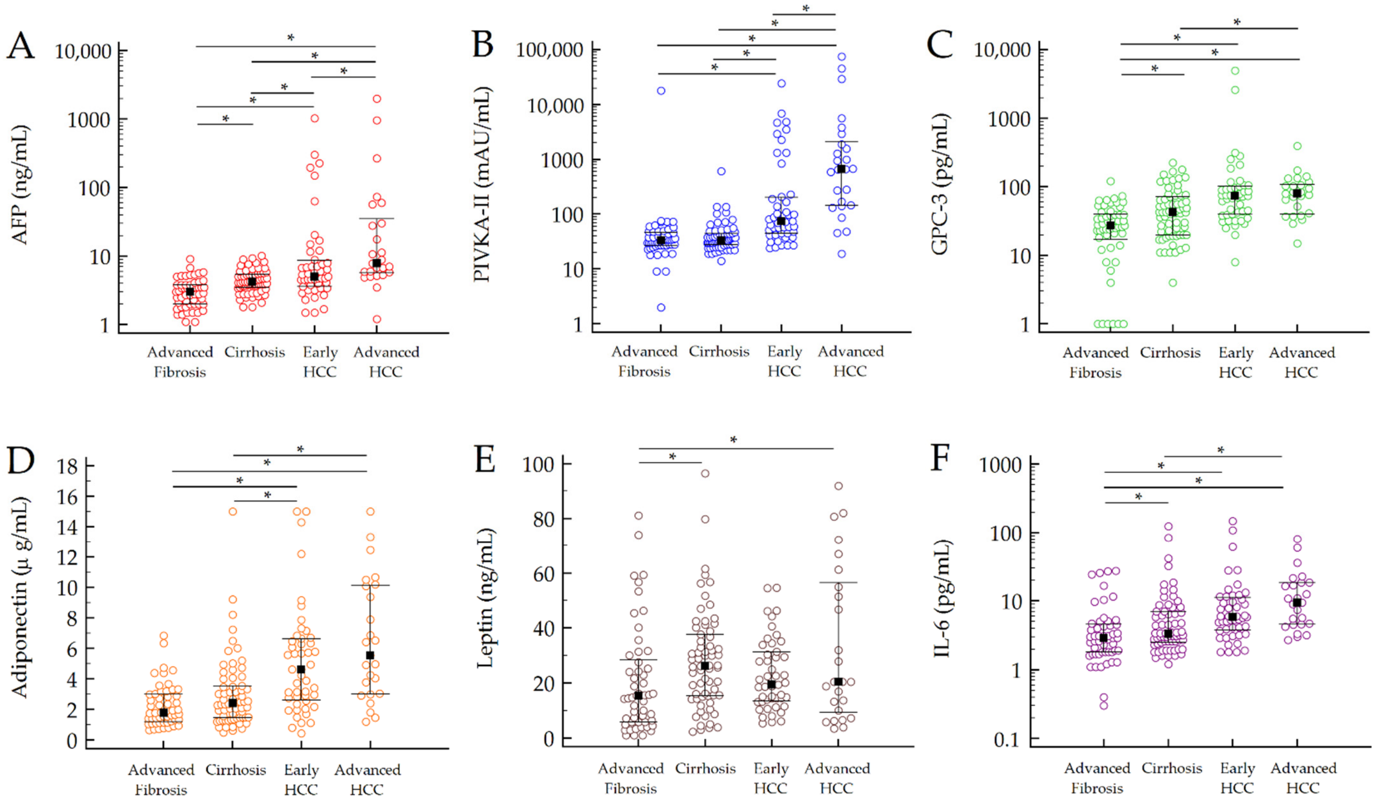

Figure S1. Median values of AFP (**A**), PIVKA-II (**B**), GPC-3 (**C**), adiponectin (**D**), leptin (**E**) and IL-6 (**F**) according to the different stages of liver disease. *$p < 0.05$; *p* values have been calculated by Mann-Whitney test. Black squares and error bars represent respectively the median value and the IQR in each group of patients. The values of AFP, PIVKA-II, GPC-3 and IL-6 have been depicted in Log scale due to data skewness. Abbreviations–alpha-fetoprotein (AFP), Barcelona Clinic Liver Cancer (BCLC), glypican 3 (GPC3), hepatocellular carcinoma (HCC), interquartile range (IQR), interleukin-6 (IL-6), protein induced by vitamin K absence or antagonist II (PIVKA-II).

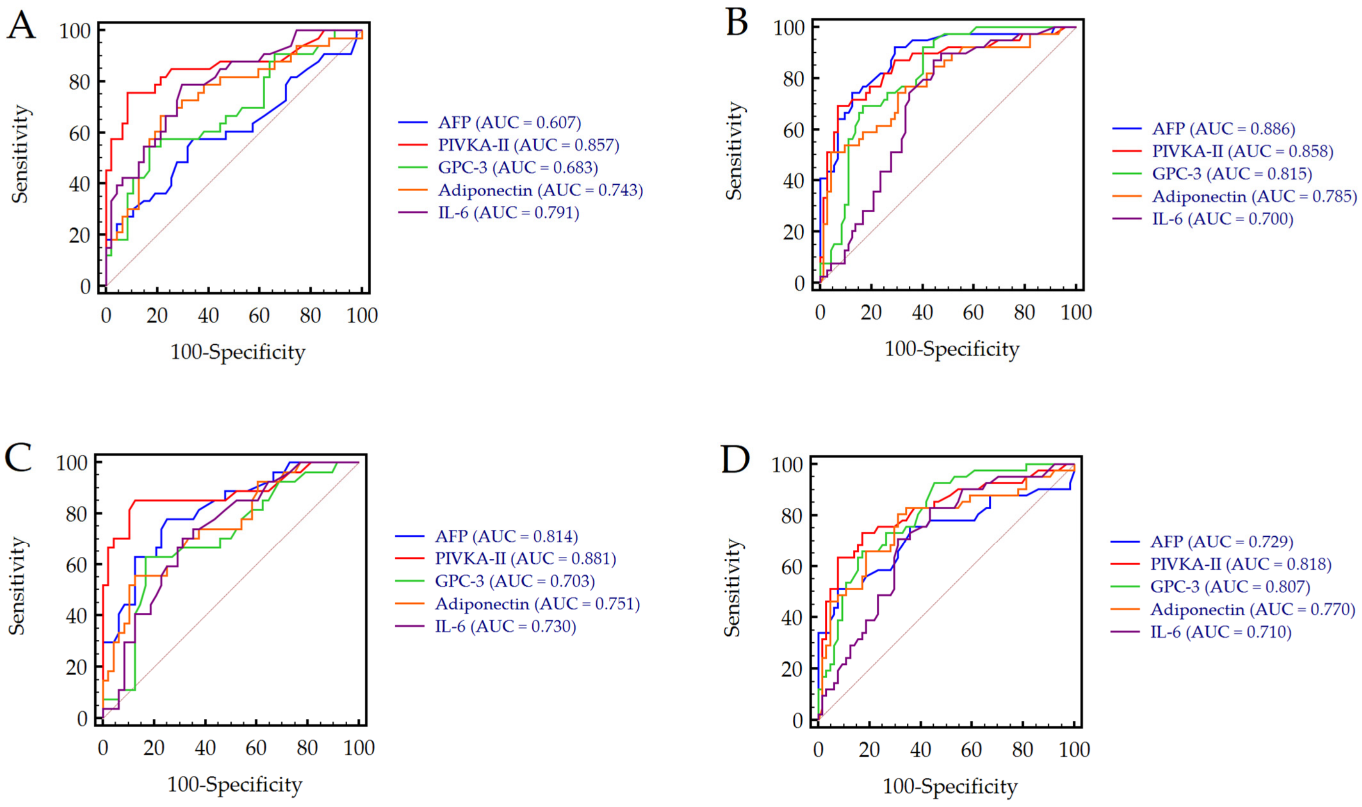

Figure S2. Diagnostic accuracy of AFP, PIVKA-II, GPC-3, adiponectin and IL-6 for the detection of HCC in lean (**A**) and obese patients (**B**), and in normal-glucose tolerant (**C**) and diabetic patients (**D**). Abbreviations–alpha-fetoprotein (AFP), glypican 3 (GPC3), hepatocellular carcinoma (HCC), interleukin-6 (IL-6), protein induced by vitamin K absence or antagonist-II (PIVKA-II).

Table S2. Comparison of the diagnostic accuracy of AFP, PIVKA-II, GPC-3, adiponectin and IL-6 for the detection of HCC.

**Biomarkers**	**AFP**	**PIVKA-II**	**GPC-3**	**Adiponectin**	**IL-6**
AFP		ΔAUC = 0.090	ΔAUC = 0.004	ΔAUC = 0.007	ΔAUC = 0.032
		$p = 0.044$	$p = 0.918$	$p = 0.888$	$p = 0.513$
PIVKA-II	ΔAUC = 0.090		ΔAUC = 0.094	ΔAUC = 0.083	ΔAUC = 0.122
	$p = 0.044$		$p = 0.035$	$p = 0.075$	$p = 0.009$
GPC-3	ΔAUC = 0.004	ΔAUC = 0.094		ΔAUC = 0.011	ΔAUC = 0.028
	$p = 0.918$	$p = 0.035$		$p = 0.818$	$p = 0.575$
Adiponectin	ΔAUC = 0.007	ΔAUC = 0.083	ΔAUC = 0.011		ΔAUC = 0.039
	$p = 0.888$	$p = 0.075$	$p = 0.818$		$p = 0.423$
IL-6	ΔAUC = 0.032	ΔAUC = 0.122	ΔAUC = 0.028	ΔAUC = 0.039	
	$p = 0.513$	$p = 0.009$	$p = 0.575$	$p = 0.423$	

Abbreviations–alpha-fetoprotein (AFP), glypican 3 (GPC3), interleukin-6 (IL-6).

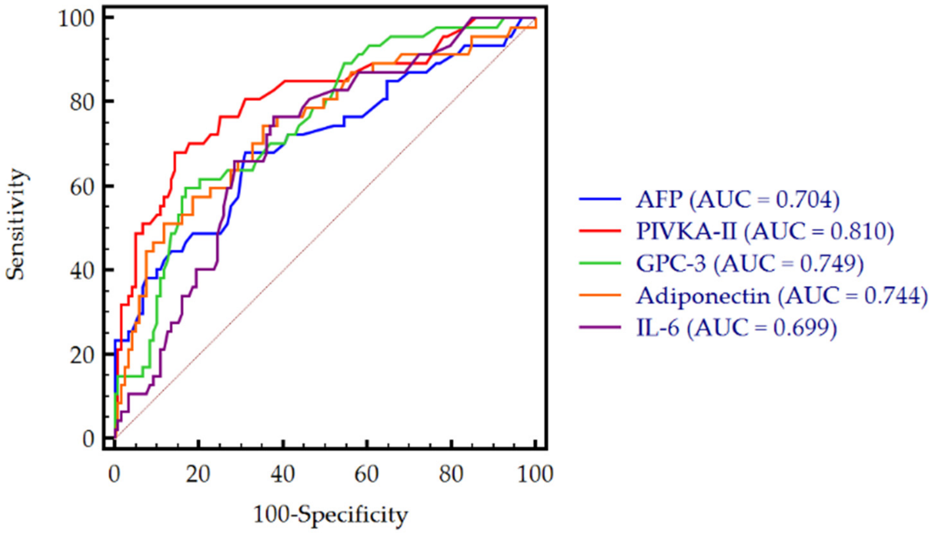

Figure S3. Diagnostic accuracy of AFP, PIVKA-II, GPC-3, adiponectin and IL-6 for the detection of early HCC. Abbreviations–alpha-fetoprotein (AFP), glypican 3 (GPC3), hepatocellular carcinoma (HCC), interleukin-6 (IL-6), protein induced by vitamin K absence or antagonist-II (PIVKA-II).
